# Identification of genetic markers of resistance to Artemisinin Combination Therapy in the rodent model *Plasmodium chabaudi*

**DOI:** 10.1186/1475-2875-9-S2-O26

**Published:** 2010-10-20

**Authors:** Louise A Rodrigues, Gisela Henriques, Sofia T Borges, Paul Hunt, Cecília P Sanchez, Axel Martinelli, Pedro Cravo

**Affiliations:** 1 Centro de Malária e Outras DoençasTropicais/IHMT/UNL, UEI Biologia Molecular, UEI Malária, Lisbon, Portugal; 2 Centre for Immunity, Infection and Evolution, School of Biological Science, The University of Edinburgh, Edinburgh, UK; 3 Hygiene Institut, Abteilung Parasitologie, Universita tsklinikum Heidelberg, Im Neuenheimer Feld 324, 69120 Heidelberg, Germany

## Background

Effective treatment of malaria relies mostly on Artemisin Combination Therapy (ACT), which consists of the administration of an Artemisinin (ART) derivative in conjunction with a chemically unrelated anti-malarial, such as Mefloquine (MF). ACTs should reduce the chances of a parasite carrying mutations conferring resistance to both drugs [1]. However, parasites resistant to the different components of the combination have recently been reported. For instance, in Southeast Asia, the appearance of parasites showing increased *in vivo* tolerance to artesunate (ATN) [2,3] may undermine the future efficacy of the ATN+MF combination.

We have previously selected stable resistance to ATN in the rodent malaria parasite *Plasmodium chabaudi*[4]. ATN-resistant parasites showed a mutation on *pcubp1* gene, coding fora deubiquitinating enzyme [5]. No changes were found in *thepcatp6 and and pcmdr1* genes [4].

In order to study the genetics of resistance to ACTs, the ATN-resistant *P.chabaudi* clone was repeatedly sub-inoculated into mice continuously treated with ATN + MF. Upon reaching a certain level of resistance, parasites were cloned by limiting dilution. The parasites' genetic background was investigated by SOLEXA whole genome re-sequencing. The identified mutations were confirmed by dideoxy sequencing real-time polymerase chain reaction.

## Results

Selection and cloning procedures originated five parasite clones, of which only one, denoted AS-ATNMF-1, was investigated. When compared to the ATN-resistant progenitor, AS-ATNMF-1 is resistant to treatment with the combination of both ATN+MF, as well as to each drug separately.

AS-ATNMF-1 carries three distinctive mutations one of which is a duplication of the *mdr1* gene. The remaining two mutations are SNPs in genes of unknown function and remain to be further investigated. No differences were found in the coding sequences of *pcatp6* and *pcubp1* when comparing AS-ATNMF-1 and its progenitor.

## Conclusions

This work provides strong evidence to support the possibility of the emergence of resistance to ACTs, even when the two drugs of the combination are administered simultaneously. Additionally, duplication of the *mdr1* gene may play a role in mediating ATN + MF resistance. However, the presence of other mutations seems to be required for the expression of this phenotype.
